# An unusual *Staphylococcus saccharolyticus* spondylodiscitis post kyphoplasty: a case report

**DOI:** 10.1186/s12879-020-05263-5

**Published:** 2020-07-23

**Authors:** Marie-Charlotte Trojani, Brigitte Lamy, Raymond Ruimy, Nicolas Amoretti, Karine Risso, Christian Roux

**Affiliations:** 1 Département de Rhumatologie, Université Cote d’Azur, CHU de Nice, Nice, France; 2 Laboratoire de Bactériologie, Hôpital L’archet 2, CHU de Nice, Nice, France; 3 INSERM U1065, Centre Méditerranéen de Médecine Moléculaire, Equipe 6, Nice, France; 4 Faculté de Médecine, Université Côte d’Azur, Nice, France; 5 Département de Radiologie, Université Cote d’Azur, CHU de Nice, Nice, France; 6 Service d’infectiologie, Université Nice Côte d’Azur, CHU de Nice, Nice, France; 7 Département de Rhumatologie, Université Cote d’Azur, LAHMESS EA6309, CNRS, iBV UMR 7277, CHU de Nice, Nice, France

**Keywords:** Spondylodiscitis, Kyphoplasty, Healthcare-associated infection, Case report, *Staphylococcus saccharolyticus*

## Abstract

**Background:**

*Staphylococcus saccharolyticus* is a rarely encountered coagulase-negative, which grows slowly and its strictly anaerobic staphylococcus from the skin. It is usually considered a contaminant, but some rare reports have described deep-seated infections. Virulence factors remain poorly known, although, genomic analysis highlights pathogenic potential.

**Case presentation:**

We report a case of *Staphylococcus saccharolyticus* spondylodiscitis that followed kyphoplasty, a procedure associated with a low rate but possible severe infectious complication (0.46%), and have reviewed the literature. This case specifically stresses the risk of healthcare-associated *S. saccharolyticus* infection in high-risk patients (those with a history of alcoholism and heavy smoking).

**Conclusion:**

*S. saccharolyticus* infection is difficult to diagnose due to microbiological characteristics of this bacterium; it requires timely treatment, and improved infection control procedure should be encouraged for high-risk patients.

## Background

*Staphylococcus saccharolyticus* (formerly known as *Peptococcus saccharolyticus*) is a rarely encountered coagulase-negative staphylococcus and the only anaerobic species of the genus *Staphylococcus* [1]. Although it is usually considered a non-pathogenic microorganism of the human skin flora with no particular known tropism to generate specific infections, occasional reports suggest a pathogenic potential through miscellaneous rare deep-seated infections [2–5]. Little is known on its virulence factors, pathogenesis, and determinants of infection. Recently, genome-sequencing analysis has shown that *S. saccharolyticus* possesses hyaluronidase activity (similar to that of *Staphylococcus aureus*), toxins of the phenol-soluble modulin family, and several quorum-sensing systems that may have a tissue-invasive potential [6].

Infectious complications after vertebroplasty/kyphoplasty are rare, but potentially serious life-threatening complications affecting the patient’s functional prognosis can occur (0.46% prevalence rate), which usually result from direct inoculation from skin flora such as *Staphylococcus aureus*, *S. epidermidis*, and *Cutibacterium acnes* [7, 8].. Here, we report the third case of spondylodiscitis due to *S. saccharolyticus* and the first to follow a surgical procedure such as kyphoplasty that specifically stresses the risk of healthcare-associated *S. saccharolyticus* infection [2, 3].

## Case presentation

**Fig. 1:**
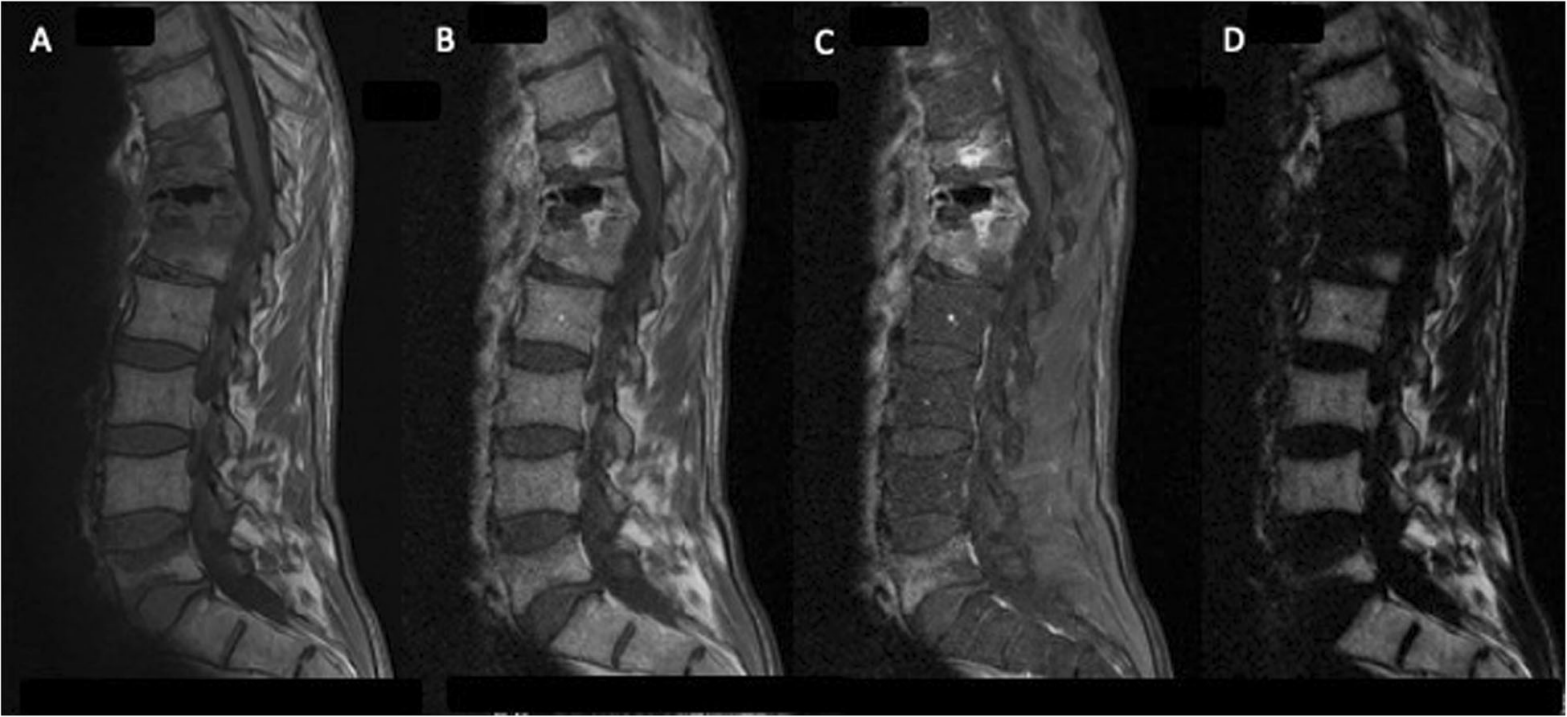
Spinal MRI, sagittal section: Hyperintensity, T11-T12-L1 vertebrae, para-vertebral soft tissue, and T12-L1 disc consistent with infectious spondylodiscitis. **a** T1-weighted; **b, c**, **d** IDEAL sequence

**Fig. 2:**
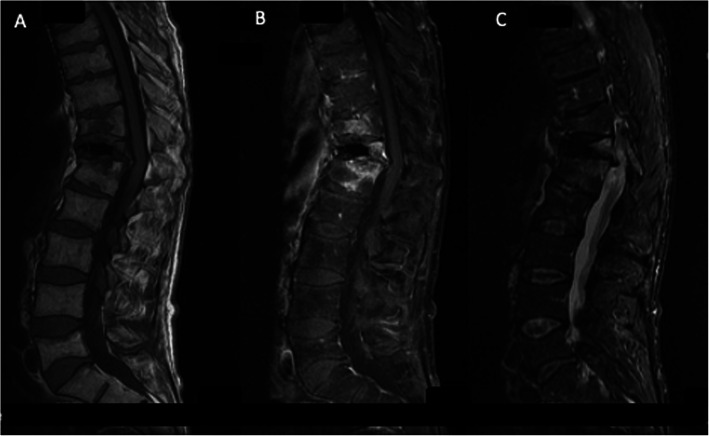
Spinal MRI, sagittal section 1 year later: A decrease in signal was observed. **a** T1-weighted; **b** T1-weighted with contrast agent; **c** STIR sequence

A 57-year-old man with a history of alcoholism and heavy smoking was admitted for disabling back pain. Four months earlier, he had sustained two vertebral fractures (T10 and T11) due to falling; these were treated by kyphoplasty under computer tomography (CT) guidance. Because the back pain persisted 2 weeks after the procedure, he received a zygapophyseal joint steroid injection under CT guidance. Three days later, his C-reactive protein level was 12.5 mg/l and hyperleukocytosis was moderate (13 G/L including 10 G/L neutrophils) and the patient had no fever. Magnetic resonance imaging (MRI) findings revealed infectious spondylodiscitis (Fig. 1). A *Staphylococcus saccharolyticus* isolate was recovered after 90 h of incubation from one single vial of a first series of three blood culture (BC) sets. The same microorganism was identified from two additional BC series collected 5 and 10 days later after 83 and 100 h of incubation, respectively. It was not possible to perform either culturing or molecular diagnostics using the tissue sample as the patient’s condition did not permit disc biopsy. However, the findings from the blood culture tests indicated a definite diagnosis of spondylodiscitis, though a catheter or spinal device was not inserted in the patient. Using disk diffusion assay, the isolate was multi-drug susceptible including to penicillin and cefoxitin. The patient was treated with 2 g of amoxicillin three times a day for a total duration of 4 weeks after consultation with the infectious disease team. Pain and inflammatory syndrome both gradually regressed, and MRI performed after 12 month showed decrease in hyperintensity (Fig. 2b). No clear source of the bacteria was identified. The infection was presumably from skin and the bacteria was likely introduced in the surgical site during the kyphoplasty procedure. However, we could not identify any defect in the surgical skin preparation and infection control procedures as well as any particular event causing exposure during the kyphoplasty procedure.

## Discussion and conclusions

*S. saccharolyticus* is a lesser known coagulase-negative staphylococcus [1]. Although it is usually considered a skin contaminant, it can cause endocarditis, bone infection, or pyomyositis, which may be associated with poor outcome (Table 1) [2–5, 9–13]. In addition to the tissue-invasive factors that have been unraveled, the dependence on anaerobic conditions is considered to favor the ability to invade human tissue, while biofilm production may contribute to the colonization of medical devices [6]. Further research is needed to understand *S. Saccharolyticus* virulence and the risk of infection better. In this regard, reports should also carefully consider patient comorbidities as host condition may contribute to the disease development. In this context, a literature review was conducted using the key words “*Staphylococcus saccharolyticus*” and “case” on the PubMed database. Eight articles were excluded after reading title and/or abstract because they were not applicable to the topic. A total of 8 articles were selected, which were used to compose the summary table (Table 1). Thus far, only 3 of the 9 reported cases have detailed host risk factors. To date, only 3 cases, including our case, of spondylodiscitis have been reported, of which 1 case has been related to a surgical procedure and multiple-level diskographies [3] and 1 (current) case occurred after kyphoplasty; information on surgical procedure was not retrieved for the third case [2].

**Table 1:** Summary of cases of infections caused by *S. saccharolyticus*

Year (ref)	Location	Age/Sex	Diagnosis	Risk factors	Comment on clinical presentation	Biology	Microbiological diagnosis	Antimicrobial susceptibility	Final treatment (total duration)	Outcome
1990 [4]	USA	61/M	Endocarditis	No predisposing valvular heart disease	Low grade fever at onset; moderate-sized mitral valve vegetation	Anemia; thrombocytosis; ESR elevation	BC; all bottles positive at day 10	Susceptible to PE, OX, VA, GE, CI, CL; resistant to ME	NAF + GE (6 wks)	Favorable at day 30
1996 [9]	USA	57/W	Prosthetic mitral valve endocarditis	NA	Fever; large masses at prosthetic valve level	Anemia; hyperleukocytosis	BC; all anaerobic vials positive at day 1; aerobic vials positive at day 11	Susceptible to VA, CL, CH; resistant to all β-lactamin agents (including OX, CES, ME, TET)	Valve change; medical treatment (NA)	Died at day 32 of hospitalization
2009 [10]	USA	NA	Prosthetic valve endocarditis	NA	NA	NA	Mitral valve; anaerobic culture; at day NA	NA	NA	NA
1990 [11]	China	21/M	Pneumonia	NA	Blood-stained sputum; multiple spherical focal lesions in the lung (CT scan)	Anemia; hyperleukocytosis	NA	Susceptible to LE, MO; NA for other antibiotics	AZ (6 d); TI + PE (3 d); IM + TI (1 d)	Died at day 120 of hospitalization
2015 [12]	China	26/W	Bone marrow infection	NA	High-grade fever; headache at onset; lymph nodes	Anemia; hyperleukocytosis; ESR and CRP strong elevation	Lung biopsy; positive anaerobic culture at day 10?	Susceptible to VA, LE, PE, CL; resistant to ME;	PE + VA (2 d); IM + VA (NA)	Died at day 114
2005 [2]	France	58/M	Spondylodiscitis	No endocarditis; no underlying disease but poor oral hygiene	Thoracic posterior pains for 2 months; fever; weight loss; NSAIDs/corticosteroids treatment	At admission, hyperleukocytosis; ESR and CRP elevation	BC and bone marrow; positive anaerobic cultures at day 3	Susceptible to VA, TEI, RI, ER, PR, TET, OF, CL; Resistant to ME; No β-lactamase production; no mecA gene	OF+CL (12 wks)	Favorable at year 1
2009 [3]	USA	38/M	Spondylodiscitis	NA	Radicular symptoms treated unsuccessfully by microdiscectomy	Elevation of inflammatory parameters	Negative aerobic cultures; negative acid-fast bacilli	NA	NA	NA
2017 [5]	NZ	48/M	Pyomyositis, spermatic cord infection	Type II diabetes; hyperlipidemia	Fever	Neutrophilia; CRP large increase; CPK normal	Multiple muscle biopsies; anaerobic positive culture at 24 h; coinfection *S. capitis* and *S. saccharolyticus*	Susceptible to FL; Resistant to PE	CEFA (1 wk); CEP (2 wks)	Favorable at 4 weeks
2017 (our case)	France	57/M	Spondylodiscitis	Heavy smoking; alcoholism; unhealthy underweight	Vertebral fractures (treated by kyphoplasty and zygapophyseal joint steroid injection); no fever; unremarkable clinical examination	Hyperleukocytosis; CRP moderate increase	Aerobic cultures negative at day 7	Susceptible to PE; CEF, MA, RI, TET, FO, OF; no β-lactamase production	AM (4 wks)	Favorable at 46 months

*M* man, *W* woman, *ESR* erythrocyte sedimentation rate, *BC* blood culture, *NA* non-available, *AM* amoxicillin, *AZ* azithromycin, *CEFA* cefazolin, *CEF* cefoxitin, *CEP* cephalexin, *CES* cephalosporin, *CH* chloramphenicol, *CI* ciprofloxacin, *CL* clindamycin, *ER* erythromycin, *FL* flucloxacillin, *FO* fosfomycin, *GE* gentamicin, *IM* imipenem, *LE* levofloxacin, *MA* macrolides, *ME* metronidazole, *MO* moxifloxacin, *NAF* nafcillin, *OF* ofloxacin, *OX* oxacillin, *PE* penicillin, *PR* pristinamycin, *RI* rifampicin, *TEI* teicoplanin, *TET* tetracycline, *TI* timidazole, *VA* vancomycin

Although vertebroplasty is a minimally invasive procedure, the possibility of postoperative infection should not be ignored. It requires major salvage surgery and may lead to residual disability and even death in several cases. In addition to standard skin preparation and the administration of prophylactic antibiotics, surgeons should preoperatively consider immune status, urinary tract infection or other infection source within 6 months, and history of pulmonary tuberculosis to prevent infection post vertebroplasty [14].

It is unclear why *S. saccharolyticus* is specifically associated with spondylodiscitis. This either reflects a specific bacterial niche that remains to be evidenced or represents a publication bias. To date, there is no means to clarify this point. Bruggeman et al. recently reported 8 strains recovered from hip and shoulder prosthetic infections, which suggests that orthopedic sites other than the spine may be infected by *S. saccharolyticus*. Unfortunately, Brüggeman et al. provided no information about the clinical cases, so it is unclear if this potential contaminant was actually the causative agent of all the reported infections [6].

This case highlights several important considerations in *S. saccharolyticus* infection and the pitfalls associated with the diagnostic aspects. Symptoms and biological syndrome may be moderate or absent in the early stage of infection [4]. In our case, fever was absent and the inflammatory biologic syndrome was mild. Possible reasons could be the proximity of a corticosteroid injection and effective empirical treatment that was timely administered. *S. saccharolyticus*, in addition to being anaerobic, grows slowly, which may be misinterpreted as a contamination because the bacterium grows only in anaerobic bottles (not in aerobic bottles); thus, very few or only a single bottle may be positive. In addition, the long time to positivity is usually a criterion to suspect BC contamination (together with a single/low number of positive bottle). Thus, the characteristics of the result (long time to positivity, low number of positive bottles) could be misinterpreted for a contamination) [1, 13]. This might also lead to under diagnosis when cultures are not incubated for at least 5 days, which is a regular situation with analyses other than BC. These findings advocate for the following: i) a minimum of 5 days of anaerobic culture study when infection is strongly suspected, and no microorganism is recovered on day 3 of incubation. This implies a preferred cooperation between a rheumatologist and microbiologist to adapt and optimize the diagnostic procedures, including molecular diagnostics, when spinal infection is suspected and ii) a fine interpretation of the microbiological findings in order to prevent overlooking an infection etiology when a microorganism that is most frequently a contaminant is recovered.

The favorable evolution after appropriate antibiotics treatment is not a regular option. The rare reported infections (9 to our knowledge) have often been fatal (3 of the 7 available outcomes; Table 1). Timely treatment may be critical. Comorbidities favoring this opportunistic infection are unevenly reported: prosthetic heart valves [9, 10], poor oral hygiene (2), type II diabetes (5), to which we can importantly add tobacco use, alcoholism, and cachexia in this patient.

Finally yet importantly, infection control procedures designed to prevent infection following vertebroplasty procedure may require some improvements to achieve infection prevention in patients with poorer condition. This is a challenging goal because, to-date, no suggestions to help reduce the risk of infection are available in the literature when all actions taken have already complied with guidelines. Strategies for improvement may arise from further research on antibacterial advanced cement for kyphoplasty [15, 16] (e.g., Clarkin et al., 2011; Brauer et al., 2013), as well as from further research on improved bundle approaches. Improvements may also arise from a better understanding of the pathophysiology of surgical site infection. A step in this direction was provided by Romano-Bertrand et al. [17], who showed how disturbances of skin microbiota by antisepsis and prophylactic treatment impacted the dynamics of microbiota in deep tissues during cardiac surgery. Although this model does not exactly fit with kyphoplasty, it does clearly show that diverse bacteria may reach the surgical site during invasive procedures. Further understanding is also needed on how the patient’s condition and innate immunity may impact the response towards controlling surgical site infection development during the very first steps of invasive procedures. In conclusion, the incidence of *S. saccharolyticus* spondylodiscitis is reportedly low, but clinicians must not fail the diagnosis. We advise that any *S. saccharolyticus* culture in the context of fever and/or orthopedic pain should be cautiously reviewed before being considered a contaminant. Prompt diagnosis and treatment is essential for an improved outcome of this severe infection and overall efforts should be made in infection control during vertebroplasty.

## Data Availability

Not applicable**.** Please contact authors for data requests.

## Abbreviations

CT: Computer tomography
BC: Blood culture
MRI: Magnetic resonance imaging
